# Adverse obstetric and neonatal outcomes complicated by psychosis among pregnant women in the United States

**DOI:** 10.1186/s12884-018-1750-0

**Published:** 2018-05-02

**Authors:** Qiu-Yue Zhong, Bizu Gelaye, Gregory L. Fricchione, Paul Avillach, Elizabeth W. Karlson, Michelle A. Williams

**Affiliations:** 1 Department of Epidemiology, Harvard T.H. Chan School of Public Health, Boston, 677 Huntington Avenue, Room Kresge 502A, Boston, MA 02115 USA; 2 Division of Psychiatry and Medicine, Pierce Division of Global Psychiatry, Massachusetts General Hospital, Boston, Massachusetts USA; 3 Department of Biomedical Informatics, Harvard Medical School, Boston, Massachusetts USA; 4 Children’s Hospital Informatics Program, Boston Children’s Hospital, Boston, Massachusetts USA; 5 Division of Rheumatology, Allergy and Immunology, Brigham and Women’s Hospital, Boston, Massachusetts USA

**Keywords:** Psychosis, Schizophrenia, Affective psychosis, National (Nationwide) Inpatient Sample (NIS), Obstetric and neonatal outcomes

## Abstract

**Background:**

Adverse obstetric and neonatal outcomes among women with psychosis, particularly affective psychosis, has rarely been studied at the population level. We aimed to assess the risk of adverse obstetric and neonatal outcomes among women with psychosis (schizophrenia, affective psychosis, and other psychoses).

**Methods:**

From the 2007 – 2012 National (Nationwide) Inpatient Sample, 23,507,597 delivery hospitalizations were identified. From the same hospitalization, *International Classification of Diseases* diagnosis codes were used to identify maternal psychosis and outcomes. Adjusted odds ratios (aOR) and 95% confidence intervals (CI) were obtained using logistic regression.

**Results:**

The prevalence of psychosis at delivery was 698.76 per 100,000 hospitalizations. After adjusting for sociodemographic characteristics, smoking, alcohol/substance abuse, and pregnancy-related hypertension, women with psychosis were at a heightened risk for cesarean delivery (aOR = 1.26; 95% CI: 1.23 - 1.29), induced labor (aOR = 1.05; 95% CI: 1.02 - 1.09), antepartum hemorrhage (aOR = 1.22; 95% CI: 1.14 - 1.31), placental abruption (aOR = 1.22; 95% CI: 1.13 - 1.32), postpartum hemorrhage (aOR = 1.18; 95% CI: 1.10 - 1.27), premature delivery (aOR = 1.40; 95% CI: 1.36 - 1.46), stillbirth (aOR = 1.37; 95% CI: 1.23 - 1.53), premature rupture of membranes (aOR = 1.22; 95% CI: 1.15 - 1.29), fetal abnormalities (aOR = 1.49; 95% CI: 1.38 - 1.61), poor fetal growth (aOR = 1.26; 95% CI: 1.19 - 1.34), and fetal distress (aOR = 1.14; 95% CI: 1.10 - 1.18). Maternal death during hospitalizations (aOR = 1.00; 95% CI: 0.30 - 3.31) and excessive fetal growth (aOR = 1.06; 95% CI: 0.98 - 1.14) were not statistically significantly associated with psychosis.

**Conclusions:**

Pregnant women with psychosis have elevated risk of several adverse obstetric and neonatal outcomes. Efforts to identify and manage pregnancies complicated by psychosis may contribute to improved outcomes.

**Electronic supplementary material:**

The online version of this article (10.1186/s12884-018-1750-0) contains supplementary material, which is available to authorized users.

## Background

Psychosis is often used as a generic description of severe mental illness characterized by delusions, hallucinations, disorganized thinking and speech, and other associated cognitive and behavioral impairments interfering with the ability to meet the ordinary demands of life [1, 2]. Psychosis is comorbid with a number of mental disorders including, but not limited to, schizophrenia, schizoaffective disorders, bipolar disorders, and major depressive disorders [3]. Immediately before and after birth, due to changes in medications, sleep deprivation, alterations in hormone levels [4, 5], and the physiological demands of pregnancy which may temporarily unmask subclinical disease [6], women may be at higher risk for psychosis, resulting in substantial distress and long-term implications for the wellbeing of mothers, families, and society [5]. Knowledge concerning the adverse obstetric and neonatal outcomes among pregnant women with psychosis is necessary to provide recommendations for optimal prenatal care [7].

However, adverse obstetric and neonatal outcomes among pregnant women with psychosis have rarely been studied at the population level, and the evidence base in many areas remains poor [5, 8]. Early studies on this topic are based on small sample sizes with limited statistical power [7]. In addition, few studies have adequately accounted for confounders [5, 7]. Furthermore, the majority of previous studies have focused on schizophrenia, and little is known about the effects of affective psychosis (bipolar or major depressive disorders with psychotic symptoms) in pregnancy [9–12].

Therefore, in this study, we sought to assess the risk of adverse obstetric and neonatal outcomes for US pregnant women with prevalent psychosis, including schizophrenia, affective psychosis, and other psychoses, at the time of delivery in a population-based sample of delivery-related hospitalizations.

## Methods

### Database

The National (Nationwide) Inpatient Sample (NIS) of the Healthcare Cost and Utilization Project (HCUP), sponsored by the Agency for Healthcare Research and Quality (AHRQ), is the largest publicly available all-payer (including Medicare, Medicaid, other non-federal payers, and patients who are uninsured) inpatient health care database in the United States [13]. Approximately 1000 hospitals that represent all types of health facilities are selected for the NIS. Five hospital characteristics — geographic region, ownership, location, teaching status, and bed size — are used for stratification to create a representative sample of US hospitalizations [13]. Before 2012, a 20% stratified random sample of hospitals within the stratum defined by the aforementioned five hospital characteristics was drawn by the AHRQ [13–15]. All discharges from sampled hospitals were included. Beginning with 2012, the NIS was redesigned to improve national estimates, and within each stratum, a 20% stratified random sample of discharges from all HCUP-participating hospitals was drawn by the AHRQ [13–15]. Discharge-level sampling weights based on the sampling scheme are available to obtain national estimates [16, 17]. Approximately seven million unweighted discharges and 35 million weighted discharges nationally are recorded each year [16, 17]. The NIS does not allow linkage of patient’s records. This study is exempt from review by institutional review boards given the data are publicly available and do not contain personal identifiers. Our study conforms to the Data Use Agreement for the Nationwide Databases from the HCUP.

### Study population

Our analysis included pregnant women aged 12-55 years who were hospitalized for delivery during 2007-2012. The identification of delivery hospitalizations was predicated upon delivery-related *International Classification of Diseases*, Ninth Revision, Clinical Modification (ICD-9-CM) diagnosis and procedure codes and Diagnosis-Related Group (DRG) codes [18–20] (Additional file 1: Table S1).

### Variable specification

#### Exposure

We used the ICD-9-CM diagnosis codes from the same delivery hospitalization to identify psychosis diagnoses. We included women with any (primary or secondary) discharge diagnosis codes for psychosis in the following groups: schizophrenia (ICD-9-CM: 295.**); affective psychosis (ICD-9-CM: 296.**), and other psychoses (ICD-9-CM: 297.** - 298.**) [21–24]. In a validation study, the set of diagnosis codes has demonstrated adequate sensitivity (91%) and positive predictive value (91%) [22].

### Obstetric and neonatal outcomes

Maternal death during hospitalization was directly extracted from the NIS. Obstetric and neonatal outcomes included in this analysis were: cesarean delivery, length of stay (in days and in percentages with stays longer than 6 days) among cesarean and vaginal deliveries, induction of labor (applies to induction by cervical dilation and medical induction of labor), antepartum hemorrhage, placental abruption, postpartum hemorrhage, spontaneous delivery earlier than 37-week gestation, stillbirth, premature rupture of membranes, excessive fetal growth (applies to large-for-dates), poor fetal growth (applies to placental insufficiency and small-for-gestational-age), fetal distress (applies to fetal metabolic acidemia), and fetal abnormality affecting management of mother (including conditions in the fetus that affecting management of mother: central nervous system malformation, chromosomal abnormality, hereditary disease in family possibly affecting fetus, suspected damage to fetus from viral disease/other disease in the mother, suspected damage to fetus from drugs or radiation, decreased fetal movements, and other known or suspected fetal abnormality, not elsewhere classified) (Additional file 1**:** Table S2).

### Covariates

Covariates including age, race/ethnicity, median household income quartiles for patient zip code, expected primary payer indicating patients’ insurance payer, length of stay, and total charges were coded in the NIS. Median household income quartiles for patient zip code provided a quartile classification estimating income of residents based on the year of data collection. Other characteristics (ever smoking, alcohol/substance abuse, non-psychotic depression, pregnancy-related hypertension, pregestational diabetes, preexisting hypertension, infection, and previous cesarean delivery) were abstracted from the same delivery hospitalization record (primary or secondary discharge diagnostic codes) using ICD-9-CM diagnosis codes listed in Additional file 1**:** Table S2. Hospital characteristics were obtained directly from the NIS, including hospital region, location, bed size, and teaching status.

### Statistical analyses

Using weights provided by the datasets, we reported national estimates representing discharges from all US community hospitals. We compared the distributions of sociodemographic, baseline, and hospital characteristics between women with and without psychosis by performing Wald Chi-square and *t*-tests. We calculated odds ratios (ORs) and 95% confidence intervals (CIs) using logistic regression. We fit three consecutive models: 1) unadjusted model; 2) minimally adjusted model with adjustment for *a priori* confounders including maternal age (continuous), race/ethnicity, median household income quartiles, hospital region (Northeast, Midwest, South, West), hospital location (rural or urban), and year as potential confounders; and 3) fully adjusted model with additional adjustment for *a priori* confounders including ever smoking, alcohol/substance abuse, and pregnancy-induced hypertension. We reported adjusted odds ratios (aORs) derived from multivariable logistic regression models. All variables had <5% missing data except for race/ethnicity. We created missing indicator variables to address missing data for race/ethnicity and median household income quartiles. Total hospitalization charges were adjusted for inflation to reflect 2012 US dollars [25].

All analyses were conducted using SAS 9.4 (SAS Institute, Cary, NC, USA) and SAS-callable SUDAAN software (version 11.0.1, RTI International, Research Triangle, NC, USA). Statistical significance was set at two-sided *P*<0.05. Some computations were run on the Odyssey cluster supported by the Faculty of Arts & Sciences Division of Science, Research Computing Group at Harvard University.

### Sensitivity Analyses

To evaluate the individual effect of schizophrenia (ICD-9-CM: 295.**) and affective psychosis (ICD-9-CM: 296.**) on adverse obstetric and neonatal outcomes, we conducted two sensitivity analyses among women with schizophrenia or women with affective psychosis. The reference group in these two analyses was identical to our main analysis, i.e., women without any psychosis. Considering the small sample size, we did not perform this analysis among women with other psychoses.

Given that multiple birth was associated with a myriad of complications such as preterm labor and fetal growth restriction [26], we first restricted our analyses to singletons. In a separate analysis, we also adjusted for multiple birth in multivariable regression analyses of all pregnancies including multifetal pregnancies.

## Results

A total of 23,507,597 delivery hospitalizations were included in this study after applying the NIS sampling weights. Among the delivery hospitalizations, 164,261 hospitalizations had a diagnosis of psychosis. The prevalence of psychosis at delivery was 698.76 per 100,000 hospitalizations. More than 90% (92.98%) of our study population were women with a diagnosis of affective psychosis (schizophrenia: *n* = 14,125; affective psychosis: *n* = 152,727; and other psychoses: *n* = 2,387). The prevalence of schizophrenia, affective psychosis, and other psychoses at delivery were 60.09, 649.69, and 10.15 per 100,000 hospitalizations, respectively.

The sociodemographic characteristics are presented in Table 1. Women with psychosis (mean age = 26.58 years) were younger as compared to women without psychosis (mean age = 27.68 years). More than 40% of women with psychosis (42.02%) were in the age group of 12-24 years while only 33.27% of women without psychosis were in this age group. Regarding race/ethnicity, as compared to women without psychosis, women with psychosis were more likely to be White (56.22% vs. 43.99%) or Black (14.97% vs. 11.81%) and less likely to be Hispanic (8.66% vs. 19.48%) or Asian or Pacific Islander (1.07% vs. 4.46%). Approximately one-third of women with psychosis (33.88%) were in the lowest quartile of median household income. The majority of women with psychosis had their medical care paid by Medicare or Medicaid (69.22%). Nearly half of women (50.13%) without psychosis had private insurance as the expected primary payer while this proportion was 25.77% among women with psychosis. Approximately 28.87% of women with psychosis had diagnosis codes for smoking while only 5.05% of women without psychosis had these diagnosis codes. Compared with women without psychosis, women with psychosis were more likely to have alcohol/substance abuse (15.67% vs. 1.46%), non-psychotic depression (16.06% vs. 1.87%), pregnancy-related hypertension (10.55% vs. 7.97%), pre-gestational diabetes (2.81% vs. 0.93%), preexisting hypertension (4.66% vs. 1.99%), infection (7.42% vs. 4.69%), and previous cesarean delivery (17.70% vs. 16.30%).

**Table 1:** Baseline characteristics of women with and without psychosis at delivery hospitalizations (*N*=23,507,597)

Characteristics	Women				*P-*value
	With psychosis (*N* = 164,261)		Without psychosis (*N* = 23,343,336)		
	n	%	n	%	
Age, mean (SD), year	26.58 (1.89)		27.68 (1.73)		<0.0001
Age categories, year					
12-18	11,707	7.13	1,230,611	5.27	<0.0001
19-24	57,315	34.89	6,536,844	28.00	
25-29	43,579	26.53	6,569,694	28.14	
30-34	31,906	19.42	5,595,824	23.97	
35-39	15,497	9.43	2,758,774	11.82	
40-55	4,258	2.59	651,590	2.79	
Race					
White	92,341	56.22	10,269,706	43.99	<0.0001
Black	24,594	14.97	2,756,003	11.81	
Hispanic	14,217	8.66	4,546,581	19.48	
Asian or Pacific Islander	1,761	1.07	1,041,542	4.46	
Native American	1,118	0.68	176,796	0.76	
Other	4,115	2.51	979,229	4.19	
Missing	26,115	15.90	3,573,478	15.31	
Median household income quartiles					
Quartile 1 (poorest)	55,646	33.88	6,240,956	26.74	<0.0001
Quartile 2	43,865	26.70	5,759,232	24.67	
Quartile 3	36,093	21.97	5,646,070	24.19	
Quartile 4 (wealthiest)	24,844	15.13	5,248,090	22.48	
Missing	3,812	2.32	448,988	1.92	
Expected primary payer					
Medicare	13,505	8.22	150,033	0.64	<0.0001
Medicaid	100,197	61.00	10,016,502	42.91	
Private insurance	42,336	25.77	11,702,370	50.13	
Self-pay	3,387	2.06	735,501	3.15	
No charge	169	0.10	49,118	0.21	
Other	4,446	2.71	647,978	2.78	
Missing	221	0.13	41,835	0.18	
Smoking	47,428	28.87	1,179,021	5.05	<0.0001
Alcohol/substance abuse	25,747	15.67	340,451	1.46	<0.0001
Non-psychotic depression	26,388	16.06	436,901	1.87	<0.0001
Pregnancy-related hypertension	17325	10.55	1859437	7.97	<0.0001
Pregestational diabetes	4,609	2.81	217,748	0.93	<0.0001
Preexisting hypertension	7,659	4.66	463,822	1.99	<0.0001
Infection	12,187	7.42	1,095,394	4.69	<0.0001
Previous cesarean delivery	29,066	17.70	3,804,658	16.30	<0.0001

Individual cell counts may not add up to the global cell counts because of rounding and the differences arising from variance computations when using the discharge weights.

Percentages may not add up to 100% due to rounding or missing data.

Abbreviations: *SD* standard deviation

Table 2 shows the hospital characteristics. As compared with women without psychosis, women with psychosis at delivery were more likely to reside in the Northeast (19.98% vs. 16.13%) and Midwest region (25.55% vs. 21.33%). No statistically significant difference was found for hospital location (rural or urban) or bed size (small, medium, or large) between women with and without psychosis. A larger proportion of women with psychosis were admitted in a teaching hospital (55.16% vs. 46.71%).

**Table 2:** Characteristics of hospitals where women with and without psychosis-hospitalizations being hospitalized (*N*=23,507,597)

Characteristics	Women				*P*-value
	With psychosis (*N* = 164,261)		Without psychosis (*N* = 23,343,336)		
	n	%	n	%	
Region					
Northeast	32,827	19.98	3,764,982	16.13	<0.0001
Midwest	41,969	25.55	4,979,708	21.33	
South	57,647	35.10	8,867,733	37.99	
West	31,818	19.37	5,730,913	24.55	
Location					
Rural	18,856	11.48	2,592,608	11.11	0.64
Urban	143,867	87.58	20,544,262	88.01	
Bed size					
Small	18,298	11.14	2,503,608	10.73	0.09
Medium	38,645	23.53	6,067,531	25.99	
Large	105,781	64.40	14,565,733	62.40	
Teaching hospital	90,599	55.16	10,902,764	46.71	<0.0001

Individual cell counts may not add up to the global cell counts because of rounding and the differences arising from variance computations when using the discharge weights

Percentages may not add up to 100% due to rounding or missing data

The frequencies and ORs for obstetric and neonatal outcomes among women with and without psychosis is presented in Table 3. In the fully adjusted models, women with psychosis were more likely to undergo a cesarean delivery (aOR = 1.26; 95% CI: 1.23 - 1.29) and induction of labor (aOR = 1.05; 95% CI: 1.02 - 1.09). The mean length of stay was longer for women with psychosis than for women without psychosis (vaginal delivery: 3.08 days vs. 2.53 days; cesarean delivery: 4.29 days vs. 3.55 days). Women with psychosis were more likely than women without psychosis to experience antepartum hemorrhage (aOR = 1.22; 95% CI: 1.14 - 1.31), placental abruption (aOR = 1.22; 95% CI: 1.13 - 1.32), postpartum hemorrhage (aOR = 1.18; 95% CI: 1.10 - 1.27), premature delivery (aOR = 1.40; 95% CI: 1.36 - 1.46), stillbirth (aOR = 1.37; 95% CI: 1.23 - 1.53), premature rupture of membranes (aOR = 1.22; 95% CI: 1.15 - 1.29), and fetal abnormalities (aOR = 1.49; 95% CI: 1.38 - 1.61). No significant association was observed between psychosis and maternal death during hospitalizations (aOR = 1.00; 95% CI: 0.30 - 3.31). Offspring of women with psychosis were more likely to experience poor fetal growth (aOR = 1.26; 95% CI: 1.19 - 1.34) and fetal distress (aOR = 1.14; 95% CI: 1.10 - 1.18) as compared to offspring of women without psychosis. However, there was no evidence of a statistically significant association between psychosis and excessive fetal growth (aOR = 1.06; 95% CI: 0.98 - 1.14). Apart from the aforementioned obstetric and neonatal outcomes, women with psychosis had larger total charges ($16,332 vs. $13,710 US dollars).

**Table 3:** Obstetric and neonatal outcomes among women with and without psychosis during delivery hospitalizations (*N*=23,507,597)

Obstetric and neonatal outcomes	Women				OR (95% CI)		
	With psychosis (*N* = 164,261)		Without psychosis (*N* = 23,343,336)		Unadjusted	Adjusted^a^	Adjusted^b^
	n	%	n	%			
Death during hospitalizations	19	0.01	1,468	0.01	1.82 (0.67, 4.92)	1.31 (0.42, 4.14)	1.00 (0.30, 3.31)
Cesarean delivery	62,496	38.05	7,718,434	33.06	1.24 (1.21, 1.28)	1.31 (1.28, 1.35)	1.26 (1.23, 1.29)
Length of stay, mean (SE), day							
Vaginal birth	3.08 (0.10)		2.53 (0.01)		NA	NA	NA
Cesarean delivery	4.29 (0.07)		3.55 (0.02)		NA	NA	NA
Length of stay >6 day							
Vaginal birth	243	0.15	9,526	0.04	4.12 (3.02, 5.63)	4.03 (2.94, 5.52)	3.19 (2.56, 4.50)
Cesarean delivery	4,800	2.92	245,416	1.05	2.53 (2.35, 2.74)	2.46 (2.27, 2.66)	2.14 (1.98, 2.32)
Induction of labor	32,876	20.01	4,247,666	18.20	1.13 (1.09, 1.17)	1.05 (1.02, 1.09)	1.05 (1.02, 1.09)
Antepartum hemorrhage	4,138	2.52	356,821	1.53	1.67 (1.55, 1.79)	1.67 (1.55, 1.79)	1.22 (1.14, 1.31)
Placental abruption	3,127	1.90	246,768	1.06	1.82 (1.68, 1.97)	1.75 (1.62, 1.90)	1.22 (1.13, 1.32)
Postpartum hemorrhage	5,460	3.32	654,043	2.80	1.19 (1.11, 1.28)	1.22 (1.13, 1.31)	1.18 (1.10, 1.27)
Spontaneous delivery <37-week gestation	20,059	12.21	1,692,651	7.25	1.78 (1.71, 1.85)	1.75 (1.68, 1.82)	1.40 (1.36, 1.46)
Stillbirth	1,811	1.10	153,045	0.66	1.69 (1.52, 1.88)	1.64 (1.47, 1.82)	1.37 (1.23, 1.53)
Premature rupture of membranes	8,318	5.06	904,679	3.88	1.32 (1.25, 1.40)	1.31 (1.24, 1.39)	1.22 (1.15, 1.29)
Excessive fetal growth	3,807	2.32	609,142	2.61	0.89 (0.82, 0.96)	0.91 (0.85, 0.98)	1.06 (0.98, 1.14)
Poor fetal growth	6,668	4.06	507,269	2.17	1.91 (1.79, 2.03)	1.75 (1.65, 1.86)	1.26 (1.19, 1.34)
Fetal distress	28,282	17.22	3,342,430	14.32	1.25 (1.20, 1.29)	1.21 (1.17, 1.26)	1.14 (1.10, 1.18)
Fetal abnormalities	4,380	2.67	337,978	1.45	1.87 (1.73, 2.01)	1.83 (1.70, 1.97)	1.49 (1.38, 1.61)

Abbreviations: *SE* standard error, *OR* odds ratio, *CI* confidence interval

^a^Adjusted for maternal age (continuous), race, median household income quartiles, hospital location, hospital region, and year

^b^Further adjusted for ever smoking, alcohol/substance abuse, and pregnancy-related hypertension

In our sensitivity analysis for women with schizophrenia (Table 4), the unadjusted odds ratios were higher for the majority of the outcomes as compared to the analysis that included all women. After further adjustment for smoking, alcohol/substance abuse, and pregnancy-related hypertension, schizophrenia was statistically significantly associated with cesarean delivery (aOR = 1.16; 95% CI: 1.08 - 1.25), hospital stays of more than 6 days (cesarean delivery: aOR = 3.01; 95% CI: 1.11 - 8.13; vaginal delivery: aOR = 2.33; 95% CI: 1.86 - 2.93), placental abruption (aOR = 1.34; 95% CI: 1.06 - 1.68), premature delivery (aOR = 1.42; 95% CI: 1.26 - 1.59), premature rupture of membranes (aOR = 1.31; 95% CI: 1.12 - 1.53), fetal distress (aOR = 1.13; 95% CI: 1.03 - 1.25), and fetal abnormalities (aOR = 1.34; 95% CI: 1.08 - 1.66). However, no statistically significant associations were found of schizophrenia with death during hospitalizations (aOR = 2.23; 95% CI: 0.30 - 16.80), induction of labor (aOR = 0.98; 95% CI: 0.89 - 1.09), antepartum hemorrhage (aOR = 1.18; 95% CI: 0.96 - 1.46), postpartum hemorrhage (aOR = 1.09; 95% CI: 0.89 - 1.34), stillbirth (aOR = 1.28; 95% CI: 0.94 - 1.74), excessive fetal growth (aOR = 0.87; 95% CI: 0.66 - 1.14), or poor fetal growth (aOR = 1.10; 95% CI: 0.91 - 1.33).

**Table 4:** Obstetric and neonatal outcomes among women with and without schizophrenia during delivery hospitalizations (*N*=23,357,461)

Obstetric and neonatal outcomes	Women				OR (95% CI)		
	With schizophrenia (*N* = 14,125)		Without psychosis (*N* = 23,343,336)		Unadjusted	Adjusted^a^	Adjusted^b^
	n	%	n	%			
Death during hospitalizations	≤10 ^c^	0.04	1,468	0.01	5.15 (0.73, 36.61)	3.21 (0.45, 23.11)	2.23 (0.30, 16.80)
Cesarean delivery	5,544	39.25	7,718,434	33.06	1.31 (1.22, 1.41)	1.22 (1.13, 1.31)	1.16 (1.08, 1.25)
Length of stay, mean (SE), day							
Vaginal birth	3.55 (0.48)		2.53 (0.01)		NA	NA	NA
Cesarean delivery	4.66 (0.19)		3.55 (0.02)		NA	NA	NA
Length of stay >6 day							
Vaginal birth	25	0.18	9,526	0.04	4.73 (1.93, 11.59)	3.86 (1.58, 9.41)	3.01 (1.11, 8.13)
Cesarean delivery	547	3.87	245,416	1.05	3.34 (2.70, 4.12)	2.71 (2.19, 3.36)	2.33 (1.86, 2.93)
Induction of labor	2,444	17.30	4,247,666	18.20	0.93 (0.85, 1.03)	0.97 (0.88, 1.07)	0.98 (0.89, 1.09)
Antepartum hemorrhage	422	2.99	356,821	1.53	1.98 (1.61, 2.44)	1.74 (1.41, 2.15)	1.18 (0.96, 1.46)
Placental abruption	355	2.51	246,768	1.06	2.43 (1.93, 3.04)	2.08 (1.66, 2.62)	1.34 (1.06, 1.68)
Postpartum hemorrhage	446	3.16	654,043	2.80	1.14 (0.93, 1.39)	1.13 (0.93, 1.39)	1.09 (0.89, 1.34)
Spontaneous delivery <37-week gestation	2,022	14.32	1,692,651	7.25	2.14 (1.92, 2.39)	1.85 (1.65, 2.06)	1.42 (1.26, 1.59)
Stillbirth	192	1.36	153,045	0.66	2.09 (1.54, 2.83)	1.62 (1.19, 2.19)	1.28 (0.94, 1.74)
Premature rupture of membranes	803	5.68	904,679	3.88	1.50 (1.28, 1.76)	1.44 (1.24, 1.69)	1.31 (1.12, 1.53)
Excessive fetal growth	237	1.68	609,142	2.61	0.64 (0.49, 0.84)	0.72 (0.55, 0.95)	0.87 (0.66, 1.14)
Poor fetal growth	560	3.96	507,269	2.17	1.86 (1.54, 2.24)	1.61 (1.33, 1.94)	1.10 (0.91, 1.33)
Fetal distress	2,590	18.34	3,342,430	14.32	1.34 (1.22, 1.49)	1.22 (1.11, 1.35)	1.13 (1.03, 1.25)
Fetal abnormalities	373	2.64	337,978	1.45	1.85 (1.50, 2.28)	1.75 (1.41, 2.16)	1.34 (1.08, 1.66)

Abbreviations: *SE* standard error, *OR* odds ratio, *CI* confidence interval

^a^Adjusted for maternal age (continuous), race, median household income quartiles, hospital location, hospital region, and year

^b^Further adjusted for ever smoking, alcohol/substance abuse, and pregnancy-related hypertension

^c^HCUP privacy protection requirements do not allow the reporting of data where there are less than or equal to 10 individual records in a given cell

Compared with the main analysis (Table 3), the results were largely similar in the sensitivity analysis for women with affective psychosis (Table 5) except for excessive fetal growth. Infants born to women with affective psychosis were at increased risk for excessive fetal growth (aOR = 1.08; 95% CI: 1.00 - 1.16). The proportion of multiple birth among women with and without psychosis was 1.87% and 1.83%, respectively. Our two methods (restricting to singletons or controlling for multiple birth) in an attempt to explore the effect of multiple birth did not materially alter the reported effect estimates (Additional file 1: Table S3, Table S4).

**Table 5:** Obstetric and neonatal outcomes among women with and without affective-psychosis during delivery hospitalizations (*N*=23,507,597)

Obstetric and neonatal outcomes	Women				OR (95% CI)		
	With affective-psychosis (*N* = 152,727)		Without psychosis (*N* = 23,343,336)		Unadjusted	Adjusted^a^	Adjusted^b^
	n	%	n	%			
Death during hospitalizations	≤10 ^c^	0.01	1,468	0.01	1.96 (0.72, 5.29)	1.49 (0.47, 4.66)	1.13 (0.34, 3.74)
Cesarean delivery	58,057	38.01	7,718,434	33.06	1.24 (1.21, 1.28)	1.33 (1.29, 1.36)	1.27 (1.24, 1.30)
Length of stay, mean (SE), day							
Vaginal birth	3.09 (0.10)		2.53 (0.01)		NA	NA	NA
Cesarean delivery	4.28 (0.07)		3.55 (0.02)		NA	NA	NA
Length of stay >6 day							
Vaginal birth	219	0.14	9,526	0.04	4.00 (2.87, 5.58)	3.98 (2.84, 5.56)	3.10 (2.15, 4.47)
Cesarean delivery	4,329	2.83	245,416	1.05	2.45 (2.26, 2.66)	2.41 (2.22, 2.62)	2.11 (1.94, 2.29)
Induction of labor	31,099	20.36	4,247,666	18.20	1.14 (1.10, 1.18)	1.06 (1.02, 1.10)	1.05 (1.02, 1.09)
Antepartum hemorrhage	3,807	2.49	356,821	1.53	1.65 (1.53, 1.77)	1.67 (1.55, 1.79)	1.22 (1.14, 1.31)
Placental abruption	2,835	1.86	246,768	1.06	1.76 (1.69, 1.84)	1.72 (1.58, 1.87)	1.20 (1.11, 1.31)
Postpartum hemorrhage	5,039	3.30	654,043	2.80	1.18 (1.10, 1.27)	1.21 (1.12, 1.30)	1.17 (1.09, 1.26)
Spontaneous delivery <37-week gestation	18,506	12.12	1,692,651	7.25	1.78 (1.71, 1.85)	1.75 (1.68, 1.82)	1.41 (1.36, 1.46)
Stillbirth	1,627	1.07	153,045	0.66	1.63 (1.46, 1.82)	1.61 (1.44, 1.80)	1.35 (1.20, 1.51)
Premature rupture of membranes	7,701	5.04	904,679	3.88	1.32 (1.24, 1.40)	1.31 (1.23, 1.39)	1.22 (1.14, 1.29)
Excessive fetal growth	3,628	2.38	609,142	2.61	0.91 (0.84, 0.98)	0.93 (0.86, 1.01)	1.08 (1.00, 1.16)
Poor fetal growth	6,229	4.08	507,269	2.17	1.92 (1.80, 2.04)	1.76 (1.66, 1.87)	1.27 (1.19, 1.35)
Fetal distress	26,240	17.18	3,342,430	14.32	1.24 (1.19, 1.29)	1.21 (1.17, 1.26)	1.14 (1.10, 1.18)
Fetal abnormalities	4,067	2.66	337,978	1.45	1.86 (1.72, 2.02)	1.83 (1.69, 1.98)	1.49 (1.38, 1.61)

Abbreviations: *SE* standard error, *OR* odds ratio, CI confidence interval

^a^Adjusted for maternal age (continuous), race, median household income quartiles, hospital location, hospital region, and year

^b^Further adjusted for ever smoking, alcohol/substance abuse, and pregnancy-related hypertension

^c^HCUP privacy protection requirements do not allow the reporting of data where there are less than or equal to 10 individual records in a given cell

## Discussion

Using a nationally representative inpatient health care database, we observed that psychosis was associated with adverse outcomes including cesarean delivery, longer length of hospital stays, induction of labor, antepartum hemorrhage, placental abruption, postpartum hemorrhage, preterm delivery, and premature rupture of membranes. Infants born to mothers with psychosis were at higher risk of stillbirth, poor fetal growth, fetal distress, and fetal abnormalities. Similar results were seen among women with affective psychosis. Newborns of women with affective psychosis were also at greater risk for excessive fetal growth. After adjusting for potential confounders including smoking, alcohol/substance abuse, and pregnancy-related hypertension, schizophrenia was statistically significantly associated with cesarean delivery, hospital stays of more than 6 days, placental abruption, premature delivery, premature rupture of membranes, fetal distress, and fetal abnormalities. Schizophrenia, however, was not associated with increased risk of induction of labor and postpartum hemorrhage.

Schizophrenia presents a higher risk for several adverse obstetric and neonatal outcomes, although the evidence is inconclusive. A meta-analysis [27] of 14 early case-control studies found that offspring of mothers with schizophrenia had elevated risk of low birthweight, stillbirth, and fetal or neonatal deaths although the effect size was small (mean correlation = 0.115). Similarly, Bennedsen’s review [7] suggested that childbearing women with schizophrenia had an increased risk of low birthweight and intrauterine growth retardation. A more recent meta-analysis indicated an almost twofold higher risk of stillbirth or fetal death among offspring of women with schizophrenia [10]. Several population-based studies have produced more clear-cut evidence. In Denmark, Bennedsen et al. [28, 29] found a significantly increased risk for preterm birth (relative risk [RR] = 1.46; 95% CI: 1.19 - 1.79), low birth weight (RR = 1.57; 95% CI: 1.36 - 1.82), and small-for-gestational age (RR = 1.34, 95% CI: 1.17 - 1.53) among women with schizophrenia. Children of women with schizophrenia had a marginally, statistically significant increase in the risk of congenital malformations. No significant increased risk of stillbirth or neonatal death was reported. However, the authors did not account for socioeconomic status, smoking, substance abuse, and psychotropic medication use in their analysis. Nilsson et al. [30] observed significantly increased risk of preterm delivery (aOR = 1.4; 95% CI: 1.2 - 1.7) and low birthweight (aOR = 1.3; 95% CI: 1.1 - 1.6), and the risk was particularly high in women admitted to hospital for schizophrenia during pregnancy. Controlling for smoking and other maternal factors (single motherhood, maternal age, parity, education, country of birth, and pregnancy-induced hypertensive diseases) reduced the risk estimates markedly. Jablensky et al. [12] found that Australian women with schizophrenia were significantly more likely to have placenta abruption (aOR = 3.17; 95% CI: 1.55 - 6.49) and to give birth to infants in the lowest weight/growth decile (aOR = 1.38; 95% CI: 1.00 - 1.90) after adjusting for maternal age, marital status, parity, aboriginality, and infant sex. No significant association was found for stillbirth, neonatal death, or fetal abnormalities although adjusted models were not present. Lin et al using a population-based dataset [31] in Taiwan found that women with schizophrenia had increased risk of delivery babies with low birthweight and small-for-gestational-age, irrespective of antipsychotics use during pregnancy after adjusting for maternal and paternal socioeconomic status, parity, hypertension, diabetes, and infant sex. Within a universal healthcare system in Canada, Vigod et al. [11] reported that infants born to women with schizophrenia were at higher risk of preterm birth (aOR = 1.75; 95% CI: 1.46 - 2.08), small-for-gestational-age (aOR = 1.49; 95% CI: 1.19 - 1.86), large-for-gestational-age (aOR = 1.53; 95% CI: 1.17 - 1.99), placental abruption (aOR = 1.98, 95% CI: 1.33 - 2.96), induction of labor (aOR = 1.35, 95% CI: 1.20 - 1.52), and cesarean delivery (aOR = 1.45, 95% CI: 1.29 - 1.62) with adjustment for maternal age, income quintile, parity, infant sex, and pre-existing diabetes and hypertension. The findings from our study confirmed that women with schizophrenia are at higher risk of preterm delivery as indicated by previous studies. However, with a considerably large sample size, we found no evidence of statistically significant associations of schizophrenia with poor fetal growth or stillbirth after adjustment for confounders including maternal smoking status.

Little is known about the effects of affective psychosis on pregnancy outcomes [9]. Using a national population cohort in Sweden, MacCabe et al. found that infants born to mothers with affective psychosis had an increased risk of low birth weight (OR = 2.22; 95% CI: 1.31 - 3.76), small-for-gestational-age (OR = 2.36; 95% CI: 1.34 - 4.16), and preterm birth (OR = 2.67; 95% CI: 1.71 - 4.17). The increased risk could not be accounted for by maternal sociodemographic characteristics, parity, smoking, and pregnancy-related hypertension. Jablensky et al. [12] investigated maternal affective psychosis (bipolar disorder and unipolar depression) as separate exposure categories. After adjustment for confounders, women with bipolar disorder were at higher risk for antepartum hemorrhage (aOR = 1.60; 95% CI: 1.11 - 2.32) while women with unipolar depression had increased risk of fetal distress (aOR = 1.27; 95% CI: 1.03 - 1.56). No significant association (unadjusted) was found for stillbirth, neonatal death, or fetal abnormalities among women with bipolar disorder or unipolar depression. In the current study, significantly increased risk was found for all outcomes except for death during hospitalizations. MacCabe et al. have demonstrated that the risk for adverse outcomes was greatest in mothers receiving hospital treatment for affective psychosis during pregnancy compared to women with a lifetime history of affective psychosis or women who were admitted for affective psychosis any time before birth [9]. The fact that diagnosis codes for psychosis and obstetric and neonatal outcomes were extracted from the same delivery hospitalizations might explain these statistically robust associations. A modest statistically significant increased risk for excessive fetal growth (aOR = 1.08; 95% CI: 1.00 - 1.16) was found in our study for women with affective psychosis. Neither of these two studies [9, 12] assessed the association of affective psychosis with excessive fetal growth. However, a recent study suggests that there might be an association between schizophrenia and excessive fetal growth [11]. A higher risk of large-for-gestational-age has been found in mothers with schizophrenia [11], which might be due to *in utero* exposure to atypical antipsychotics [32] and the predisposition of women with schizophrenia to metabolic syndrome and metabolic abnormalities such as preconception diabetes or gestational diabetes [11, 33–36].This finding concerning excessive fetal growth among offspring of women with affective psychosis deserves to be investigated in future research, and possible mechanisms should be explored. Further, there may be merits to studying whether maternal psychosis is associated with increased long-term risk of metabolic syndrome in offspring.

An elevated risk for fetal abnormalities was observed for women with psychosis, schizophrenia, and affective psychosis in our analyses. The increased risk of fetal abnormalities among offspring of women with schizophrenia has been speculated to be related to the use of antipsychotics medications during pregnancy [28]. However, several reviews [37, 38] have not implicated any specific antipsychotic medication as teratogen [5]. The absence of information pertaining to maternal use of medication in the NIS prohibited our evaluation of the independent and joint associations of psychosis and medication use/withdrawal on obstetric and neonatal outcomes.

A combination of socioeconomic, behavioral, genetic factors and comorbid medical conditions, and environmental factors may explain the higher risk of adverse obstetric and neonatal outcomes among women with psychosis [8, 10]. Women with psychosis tend to live in poor socioeconomic conditions, lack social support, and have unwanted pregnancies [7, 11, 28]. They are known to have worse health behaviors, such as smoking, substance abuse, poor diet, and lack of exercise [7, 8]. In addition, psychosis has been shown to impair a patient’s ability to seek appropriate medical care, recognize physical symptoms or warning signs, and be compliant with treatment [8]. Women with psychotic disorders are less likely to seek prenatal care [39] and may avoid psychiatric care during pregnancy [8, 39]. Investigators have also speculated that women with psychosis are at increased genetic risk for developing adverse obstetric and neonatal outcomes considering its high heritability and possible neurodevelopmental origins *in utero* [7, 11, 40, 41]. Furthermore, patients with psychosis, specifically those exposed to antipsychotics, have significantly higher rates of metabolic complications such as hypertension and diabetes [8, 39]. These metabolic complications could also be present in pregnancy, manifesting in higher rates of pregnancy related hypertensive disorders and gestational diabetes [8, 11, 34]. Infection and inflammation also have a critical role in psychosis [42], which has been shown to be associated with various adverse obstetric and neonatal outcomes [43–45]. Although the causal mechanisms are not well-established, some posit that environmental factors, such as seasonality which relates to sun-derived vitamin D, might be another potential explanation for the observed associations. The main source of vitamin D is sunlight-induced synthesis in the skin [46]. Therefore, vitamin D shows a natural fluctuation throughout the year (i.e., seasonality), with maternal vitamin D insufficiency more likely to occur during winter due to the reduced winter photoperiod in regions with less winter sunlight [47–52]. Vitamin D deficiency has been linked with psychosis [48, 50, 53–56]. In addition, vitamin D regulates placental development and function, and can influence critical components of early brain development, suggesting that maternal vitamin D deficiency may be associated with adverse outcomes of pregnancy including miscarriage, preeclampsia, and preterm birth [57, 58].

In our analysis, women with psychosis were in poorer socioeconomic conditions and had a higher prevalence of smoking, alcohol/substance abuse, pregnancy-related hypertension, preexisting hypertension, pregestational diabetes, and infection. We have shown that poor socioeconomic conditions, worse health behaviors, and comorbid medical conditions were very likely to be responsible for the increased risk of adverse pregnancy outcomes among women with schizophrenia and, to a lesser degree, among women with affective psychosis [12]. However, the increased risk for adverse obstetric and neonatal outcomes was not fully explained by these factors, especially for women with affective psychosis. Future studies are needed to consider other genetic and environmental factors. Special attention should be paid to women with affective psychosis, an understudied population with increased risk of many adverse obstetric and neonatal outcomes that were not accounted for by socioeconomic and behavioral factors.

The major strengths of our study included the considerably large sample size and adjustment for important covariates including smoking, alcohol abuse, substance abuse, and pregnancy-related hypertension. The large sample size not only represented the general US pregnant population at delivery but also provided ample statistical power. We also presented separate results for affective psychosis. However, several limitations are noteworthy. The cross-sectional design limited our ability to establish causal relation and temporality between psychosis and adverse obstetric and neonatal outcomes. Although adverse pregnancy outcomes, such as perinatal death, congenital malformations, preterm birth, might be triggering factors of psychosis [24], these adverse outcomes were not likely to cause incident psychosis in our dataset considering that new-onset psychosis in pregnancy was exceptionally rare [59] and the mean length of delivery hospital stay in this study population was three days. In addition, we had no information on the time of disease onset and disease severity during pregnancy. Previous studies have suggested that the time of disease onset and case ascertainment in relation to childbirth is critical [12, 30]. Women admitted to hospital for schizophrenia during pregnancy were at particularly high risk for adverse obstetric and neonatal outcomes as compared to those who developed schizophrenia before pregnancy [12, 30]. We were not able to replicate this finding, which could be important to understand the cause of reproductive pathology in women with psychosis [12]. Also, some important reproductive characteristics were not included in the NIS dataset, including parity and gestational age. Furthermore, we used the ICD-9-CM codes to identify psychosis, obstetric and neonatal outcomes, and covariates. These diagnosis codes, which were coded for administrative and billing purposes, may not capture the quality of data desired for research purpose. For example, lifetime smoking status was defined by a set of diagnosis codes that applied to tobacco use disorder and personal history of tobacco use, which was very likely to be underreported. A lack of exact data on smoking could lead to residual confounding. Besides, the ICD-9-CM codes used to identify psychosis included psychosis in remission (1.39%), which may result in an underestimation of the associations between psychosis and adverse outcomes.

## Conclusion

In conclusion, we observed that women with psychosis had elevated risk of adverse obstetric and neonatal outcomes. Therefore, it is important to raise awareness among clinicians of the increased risk of adverse obstetric and neonatal outcomes among these women. Coordinated antepartum care involving obstetric and psychiatric services providers may optimally manage maternal psychotic symptoms and contribute to improved obstetric and neonatal outcomes among this high-risk group of patients [8]. Special attention should be paid to women with affective psychosis. Women with affective psychosis have increased risk of several adverse obstetric and neonatal outcomes that are not accounted for by socioeconomic and behavioral factors. Future research on other genetic and environmental factors is warranted to elucidate the mechanisms underlying these associations. Behavioral health and medical interventions that target modifiable risk factors, such as smoking, hypertension, and diabetes, may be indicated for pregnant women with schizophrenia [11].

## Additional file

Additional file 1: **Table S1.** International Classification of Diseases, Ninth Revision, Clinical Modification (ICD-9-CM) diagnosis and procedure codes, Diagnosis-Related Group (DRG) codes used to determine delivery-related hospitalizations. **Table S2.** International Classification of Diseases, Ninth Revision, Clinical Modification (ICD-9-CM) diagnosis and procedure codes used to determine selected baseline characteristics and obstetric and neonatal outcomes. **Table S3.** Obstetric and neonatal outcomes among women with and without psychosis during delivery hospitalizations (*N*=23,507,597). **Table S4.** Obstetric and neonatal outcomes among women with and without psychosis during singleton delivery hospitalizations (*N* = 23,076,251). (DOCX 19 kb)

## Abbreviations

AHRQ: Agency for Healthcare Research and Quality
aOR: Adjusted odds ratios
CI: 95% confidence intervals
DRG: Diagnosis-Related Group
HCUP: Healthcare Cost and Utilization Project
ICD-9-CM: *International Classification of Diseases*, Ninth Revision, Clinical Modification
NIS: National (Nationwide) Inpatient Sample
OR: Odds ratios

## References

[1] Gaebel W, Zielasek J (2015). Focus on psychosis. Dialogues Clin. Neurosci..

[2] Fergusson DM, Poulton R, Smith PF, Boden JM (2006). Cannabis and psychosis. BMJ..

[3] Bramon E, Pirinen M, Strange A, Lin K, Psychosis Endophenotypes International Consortium, Wellcome Trust Case-Control Consortium 2 (2014). A genome-wide association analysis of a broad psychosis phenotype identifies three loci for further investigation. Biol. Psychiatry.

[4] DeVylder J, Koyanagi A (2016). Pregnant and peripartum women are not at increased risk for psychotic experiences at the population level: Evidence from 46 countries. Schizophr. Res..

[5] Jones I, Chandra PS, Dazzan P, Howard LM (2014). Bipolar disorder, affective psychosis, and schizophrenia in pregnancy and the post-partum period. Lancet..

[6] Williams D (2003). Pregnancy: a stress test for life. Curr. Opin. Obstet. Gynecol..

[7] Bennedsen BE (1998). Adverse pregnancy outcome in schizophrenic women: occurrence and risk factors. Schizophr. Res..

[8] Gold KJ, Marcus SM (2008). Effect of maternal mental illness on pregnancy outcomes. Expert Rev. Obstet. Gynecol.

[9] MacCabe JH, Martinsson L, Lichtenstein P, Nilsson E, Cnattingius S, Murray RM (2007). Adverse pregnancy outcomes in mothers with affective psychosis. Bipolar Disord..

[10] Webb R, Abel K, Pickles A, Appleby L (2005). Mortality in offspring of parents with psychotic disorders: a critical review and meta-analysis. Am. J. Psychiatry..

[11] Vigod SN, Kurdyak PA, Dennis CL (2014). Maternal and newborn outcomes among women with schizophrenia: a retrospective population-based cohort study. BJOG..

[12] Jablensky AV, Morgan V, Zubrick SR, Bower C, Yellachich L-A (2005). Pregnancy, delivery, and neonatal complications in a population cohort of women with schizophrenia and major affective disorders. Am. J. Psychiatry..

[13] Introduction to the HCUP national inpatient sample (NIS) [Internet]. Healthcare Cost and Utilization Project (HCUP). 2015 [cited 2016 Feb 1]. Available from: https://www.hcup-us.ahrq.gov/db/nation/nis/nisarchive.jsp. Accessed 1 Feb 2016.

[14] Healthcare Cost and Utilization Project (HCUP) KID Notes [Internet]. [cited 2017 Jun 9]. Available from: https://www.hcup-us.ahrq.gov/db/vars/zipinc_qrtl/kidnote.jsp

[15] Nationwide Inpatient Sample redesign report [Internet]. Healthcare Cost and Utilization Project (HCUP). 2015 [cited 2016 Feb 1]. Available from: https://www.hcup-us.ahrq.gov/db/nation/nis/reports/NISRedesignFinalReport040914.pdf

[16] NIS Trend Weights [Internet]. Healthcare Cost and Utilization Project (HCUP). 2015 [cited 2016 Feb 1]. Available from: https://www.hcup-us.ahrq.gov/db/nation/nis/trendwghts.jsp

[17] HCUP Methods Series Calculating National Inpatient Sample (NIS) Variances for Data Years 2012 and Later [Internet]. Healthcare Cost and Utilization Project (HCUP). 2015 [cited 2016 Feb 1]. Available from: https://www.hcup-us.ahrq.gov/reports/methods/2015_09.jsp

[18] Bryant A, Mhyre JM, Leffert LR, Hoban RA, Yakoob MY, Bateman BT (2012). The association of maternal race and ethnicity and the risk of postpartum hemorrhage. Anesth. Analg..

[19] Hornbrook MC, Whitlock EP, Berg CJ, Callaghan WM, Bachman DJ, Gold R (2007). Development of an algorithm to identify pregnancy episodes in an integrated health care delivery system. Health Serv. Res..

[20] Kuklina EV, Whiteman MK, Hillis SD, Jamieson DJ, Meikle SF, Posner SF (2008). An enhanced method for identifying obstetric deliveries: implications for estimating maternal morbidity. Matern. Child Health J..

[21] Selten JP, van der Graaf Y, van Duursen R, Gispen-de Wied CC, Kahn RS (1999). Psychotic illness after prenatal exposure to the 1953 Dutch Flood Disaster. Schizophr. Res..

[22] Nazareth I, King M, Haines A, Rangel L, Myers S (1993). Accuracy of diagnosis of psychosis on general practice computer system. BMJ.

[23] Marcelis M, Navarro-Mateu F, Murray R, Selten J-P, Van Os J (1998). Urbanization and psychosis: a study of 1942–1978 birth cohorts in The Netherlands. Psychol. Med.

[24] Valdimarsdóttir U, Hultman CM, Harlow B, Cnattingius S, Sparén P (2009). Psychotic illness in first-time mothers with no previous psychiatric hospitalizations: a population-based study. PLoS Med..

[25] Consumer Price Index, 1913-|Federal Reserve Bank of Minneapolis [Internet]. [cited 2017 Nov 26]. Available from: https://www.minneapolisfed.org/community/teaching-aids/cpi-calculator-information/consumer-price-index-and-inflation-rates-1913. Accessed 26 Nov 2017.

[26] Garite TJ, Clark RH, Elliott JP, Thorp JA (2004). Twins and triplets: the effect of plurality and growth on neonatal outcome compared with singleton infants. Am. J. Obstet. Gynecol..

[27] Sacker A, Done DJ, Crow TJ (1996). Obstetric complications in children born to parents with schizophrenia: a meta-analysis of case–control studies. Psychol. Med.

[28] Bennedsen BE, Mortensen PB, Olesen AV, Henriksen TB (2001). Congenital malformations, stillbirths, and infant deaths among children of women with schizophrenia. Arch. Gen. Psychiatry..

[29] Bennedsen BE, Mortensen PB, Olesen AV, Henriksen TB (1999). Preterm birth and intra-uterine growth retardation among children of women with schizophrenia. Br. J. Psychiatry..

[30] Nilsson E, Lichtenstein P, Cnattingius S, Murray RM, Hultman CM (2002). Women with schizophrenia: pregnancy outcome and infant death among their offspring. Schizophr. Res..

[31] Lin H-C, Chen I-J, Chen Y-H, Lee H-C, Wu F-J (2010). Maternal schizophrenia and pregnancy outcome: Does the use of antipsychotics make a difference?. Schizophr. Res..

[32] Newham JJ, Thomas SH, MacRitchie K, McElhatton PR, McAllister-Williams RH (2008). Birth weight of infants after maternal exposure to typical and atypical antipsychotics: prospective comparison study. Br. J. Psychiatry..

[33] Mitchell AJ, Vancampfort D, Sweers K, van Winkel R, Yu W, De Hert M (2013). Prevalence of metabolic syndrome and metabolic abnormalities in schizophrenia and related disorders--a systematic review and meta-analysis. Schizophr. Bull..

[34] Bodén R, Lundgren M, Brandt L, Reutfors J, Kieler H (2012). Antipsychotics during pregnancy: relation to fetal and maternal metabolic effects. Arch. Gen. Psychiatry..

[35] Ahlsson F, Lundgren M, Tuvemo T, Gustafsson J, Haglund B (2010). Gestational diabetes and offspring body disproportion. Acta Paediatr..

[36] Baird JD (1969). Some aspects of carbohydrate metabolism in pregnancy with special reference to the energy metabolism and hormonal status of the infant of the diabetic woman and the diabetogenic effect of pregnancy. J. Endocrinol..

[37] Gentile S (2010). Antipsychotic therapy during early and late pregnancy. A systematic review. Schizophr. Bull..

[38] Galbally M, Snellen M, Power J (2014). Antipsychotic drugs in pregnancy: a review of their maternal and fetal effects. Ther Adv Drug Saf..

[39] Howard LM (2005). Fertility and pregnancy in women with psychotic disorders. Eur. J. Obstet. Gynecol. Reprod. Biol..

[40] Mittal VA, Ellman LM, Cannon TD (2008). Gene-environment interaction and covariation in schizophrenia: the role of obstetric complications. Schizophr. Bull..

[41] Suvisaari JM, Taxell-Lassas V, Pankakoski M, Haukka JK, Lönnqvist JK, Häkkinen LT (2013). Obstetric complications as risk factors for schizophrenia spectrum psychoses in offspring of mothers with psychotic disorder. Schizophr. Bull..

[42] Yolken RH, Torrey EF (2008). Are some cases of psychosis caused by microbial agents? A review of the evidence. Mol. Psychiatry..

[43] Gibbs RS (2001). The relationship between infections and adverse pregnancy outcomes: an overview. Ann. Periodontol..

[44] Liu B, Roberts CL, Clarke M, Jorm L, Hunt J, Ward J (2013). Chlamydia and gonorrhoea infections and the risk of adverse obstetric outcomes: a retrospective cohort study. Sex. Transm. Infect..

[45] Ellis J, Williams H, Graves W, Lindsay MK (2002). Human immunodeficiency virus infection is a risk factor for adverse perinatal outcome. Am. J. Obstet. Gynecol..

[46] Boyle VT, Thorstensen EB, Mourath D, Jones MB, McCowan LME, Kenny LC, et al. The relationship between 25-hydroxyvitamin D concentration in early pregnancy and pregnancy outcomes in a large, prospective cohort. Br J Nutr. 2016;116:1409–15. 10.1017/S0007114516003202 27753425

[47] McGrath J. Hypothesis: is low prenatal vitamin D a risk-modifying factor for schizophrenia? Schizophr Res. 1999;40:173–7. 10.1016/s0920-9964(99)00052-3 10638855

[48] Bruins J, Jörg F, van den Heuvel ER, Bartels-Velthuis AA, Corpeleijn E, Muskiet FAJ, et al. The relation of vitamin D, metabolic risk and negative symptom severity in people with psychotic disorders. Schizophr. Res [Internet]. 2017; Available from: http://dx.doi.org/10.1016/j.schres.2017.08.059. 10.1016/j.schres.2017.08.059 28927862

[49] Rosecrans R, Dohnal JC. Seasonal vitamin D changes and the impact on health risk assessment. Clin Biochem. 2014;47:670–2. 10.1016/j.clinbiochem.2014.02.004 24525255

[50] Byrne EM, Psychiatric Genetics Consortium Major Depressive Disorder Working Group, Raheja UK, Stephens SH, Heath AC, Madden PAF, et al. Seasonality shows evidence for polygenic architecture and genetic correlation with schizophrenia and bipolar disorder. J Clin Psychiatry. 2015;76:128–34. 10.4088/JCP.14m08981 PMC4527536 25562672

[51] Chirumbolo S, Bjørklund G, Sboarina A, Vella A. The Role of Vitamin D in the Immune System as a Pro-survival Molecule. Clin Ther. 2017;39:894–916. 10.1016/j.clinthera.2017.03.021 28438353

[52] Daglar K, Tokmak A, Kirbas A, Guzel AI, Erkenekli K, Yucel A, et al. Maternal serum vitamin D levels in pregnancies complicated by neural tube defects. J Matern Fetal Neonatal Med. 2016;29:298–302. 10.3109/14767058.2014.999037 25544605

[53] Eyles DW, Burne THJ, McGrath JJ. Vitamin D, effects on brain development, adult brain function and the links between low levels of vitamin D and neuropsychiatric disease. Front Neuroendocrinol. 2013;34:47–64. 10.1016/j.yfrne.2012.07.001 22796576

[54] Crews M, Lally J, Gardner-Sood P, Howes O, Bonaccorso S, Smith S, et al. Vitamin D deficiency in first episode psychosis: a case-control study. Schizophr Res. 2013;150:533–7. 10.1016/j.schres.2013.08.036 24060571

[55] Schneider B, Weber B, Frensch A, Stein J, Fritz J. Vitamin D in schizophrenia, major depression and alcoholism. J Neural Transm. 2000;107:839–42. 10.1007/s007020070063 11005548

[56] Belvederi Murri M, Respino M, Masotti M, Innamorati M, Mondelli V, Pariante C, et al. Vitamin D and psychosis: mini meta-analysis. Schizophr. Res. 2013;150:235–9. 10.1016/j.schres.2013.07.017 23906618

[57] Bodnar LM, Simhan HN, Powers RW, Frank MP, Cooperstein E, Roberts JM. High prevalence of vitamin D insufficiency in black and white pregnant women residing in the northern United States and their neonates. J Nutr. 2007;137:447–52. 10.1093/jn/137.2.447 PMC4288960 17237325

[58] Evans KN, Bulmer JN, Kilby MD, Hewison M. Vitamin D and placental-decidual function. J Soc Gynecol Investig. 2004;11:263–71. 10.1016/j.jsgi.2004.02.002 15219879

[59] Wilson MP, Nordstrom K, Shah AA, Vilke GM. Psychiatric Emergencies in Pregnant Women. Emerg Med Clin North Am. 2015;33:841–51. 10.1016/j.emc.2015.07.010 26493527

