# Supplementary material for: Adverse obstetric and neonatal outcomes complicated by psychosis among pregnant women in the United States
Source: BMC Pregnancy Childbirth. 2018 May 2;18:120. doi: 10.1186/s12884-018-1750-0 (PMC5930732; doi:10.1186/s12884-018-1750-0)
Supplement: Supplementary file 1 — Table S1. International Classification of Diseases, Ninth Revision, Clinical Modification (ICD-9-CM) diagnosis and procedure codes, Diagnosis-Related Group (DRG) codes used to determine delivery-related hospitalizations. Table S2. International Classification of Diseases, Ninth Revision, Clinical Modification (ICD-9-CM) diagnosis and procedure codes used to determine selected baseline characteristics and obstetric and neonatal outcomes. Table S3. Obstetric and neonatal outcomes among women with and without psychosis during delivery hospitalizations (N=23,507,597). Table S4. Obstetric and neonatal outcomes among women with and without psychosis during singleton delivery hospitalizations (N = 23,076,251). (DOCX 19 kb) [file 12884_2018_1750_MOESM1_ESM.docx]

**Supplement Table 1. International Classification of Diseases, Ninth Revision, Clinical Modification (ICD-9-CM) diagnosis and procedure codes, Diagnosis-Related Group (DRG) codes used to determine delivery-related hospitalizations**

| **Inclusion criteria** | |
| --- | --- |
| ICD-9-CM Diagnosis codes: | |
| V27.** | Outcome of delivery |
| 650.** | Normal Delivery |
| ICD-9-CM Procedure codes: | |
| 72.** | Forceps, vacuum, and breech delivery |
| 73.22 | Internal and combined version with extraction |
| 73.59 | Other manually assisted delivery |
| 73.6* | Episiotomy |
| 74.0* | Classical cesarean section |
| 74.1* | Low cervical cesarean section |
| 74.2* | Extra peritoneal cesarean section |
| 74.4* | Cesarean section of other specified type |
| 74.99 | Other cesarean section of unspecified type |
| DRG codes | |
| 370 | Cesarean section with complications, comorbidities |
| 371 | Cesarean section without complications, comorbidities |
| 372 | Vaginal delivery with complicating diagnoses |
| 373 | Vaginal delivery without complicating diagnoses |
| 374 | Vaginal delivery with sterilization&/or dilation & curettage |
| 375 | Vaginal delivery with operating room procedure except sterilization &/or dilation& curettage |
| **Exclusion criteria** | |
| ICD-9-CM Diagnosis codes: | |
| 630.** - 639.** | Ectopic and molar pregnancy and other pregnancy with abortive outcome |
| ICD-9-CM Procedure codes: | |
| 69.01 | Dilation and curettage for termination of pregnancy |
| 69.51 | Aspiration curettage of uterus for termination of pregnancy |
| 74.91 | Hysterectomy to terminate pregnancy |
| 75.0 | Intra-amniotic injection for abortion |

**Supplement Table 2. International Classification of Diseases, Ninth Revision, Clinical Modification (ICD-9-CM) diagnosis and procedure codes used to determine selected baseline characteristics and obstetric** **and neonatal outcomes**

| **ICD-9-CM Diagnosis codes** |  |
| --- | --- |
| Ever smoking | 305.1*, V15.82, 649.0* |
| Alcohol/substance abuse | 291.**, 292.**, 303.**, 304.**, 305.**, 648.3*, 655.5*, 965.0*, V65.42 |
| Non-psychotic depression | 296.2*, 296.3*, 296.82, 300.4*, 301.12, 309.0*, 309.1*, 309.28, 311.** |
| Pregnancy-related hypertension | 642.3*, 642.4*, 642.5*, 642.6*, 642.7*, 642.9* |
| Pregestational diabetes | 250.**, 648.0* |
| Preexisting hypertension | 642.0*, 642.1*, 642.2*, 642.7*, 401.**, 402.**, 403.**, 404.**, 405.** |
| Infection | 001.**, 002.**, 003.**, 004.**, 005.**, 008.**, 009.**, 013.**, 018.**, 020.**, 021.**, 022.**, 023.**, 024.**, 025.**, 026.**, 027.**, 032.**, 033.**, 034.**, 035.**, 036.**, 037.**, 038.**, 039.**, 040.**, 041.**, 098.**, 100.**, 101.**, 112.**, 114.**, 115.**, 116.**, 117.**, 118.**, 320.**, 321.**, 324.**, 325.**, 360.**, 376.**, 383.**, 421.**, 461.**, 462.**, 463.**, 464.**, 465.**, 475.**, 481.**, 482.**, 485.**, 486.**, 494.**, 510.**, 513.**, 540.**, 541.**, 542.**, 566.**, 567.**, 572.**, 575.**, 590.**, 599.**, 601.**, 604.**, 614.**, 615.**, 670.**, 681.**, 682.**, 683.**, 685.**, 686.**, 711.**, 730.**, 790.7*, 958.3*, 996.6*, 998.5*, 999.3*, 675.1*, 658.4*, 646.6*, 616.4*, 616.3*, 572.1*, 569.5*, 522.5*, 522.7*, 526.4*, 527.3*, 528.3*, 728.86, 569.83, 569.61, 562.13, 562.11, 562.03, 562.01, 491.21, 420.99, 380.14 |
| Previous cesarean delivery | 654.2* |
| Multiple birth | V27.2* - V27.7*, 651.** |
| Vaginal delivery | 72.0*-72.4*, 72.7* |
| Cesarean delivery | 699.7* |
| Antepartum hemorrhage | 641.1*, 641.2*, 641.3*, 641.8*, 641.9* |
| Placental abruption | 641.2* |
| Postpartum hemorrhage | 666.0*, 666.1*, 666.2*, 666.3* |
| Spontaneous delivery < 37-week gestation | 644.2* |
| Stillbirth | 656.4*, V27.1*, V27.3*, V27.4*, V27.6*, V27.7* |
| Premature rupture of membranes | 658.10, 658.11, 658.13 |
| Excessive fetal growth | 656.6* |
| Poor fetal growth | 656.5* |
| Fetal distress | 656.3* |
| Fetal abnormality affecting management of mother | 655.** |
| **ICD-9-CM Procedure codes:** |  |
| Cesarean delivery | 74.0*, 74.1*, 74.2*, 74.4*, 74.99 |
| Induction of labor | 73.1*, 73.4* |

**Supplement Table 3. Obstetric and neonatal outcomes among women with and without psychosis during delivery hospitalizations (N=23,507,597)**

| **Obstetric and neonatal outcomes** | **Women** | | | | |  | **OR (95% CI)** | |  |  |
| --- | --- | --- | --- | --- | --- | --- | --- | --- | --- | --- |
|  | **With psychosis**  **(N = 164,261)** | |  | **Without psychosis**  **(N = 23,343,336)** | |  | **Unadjusted** | **Adjusted^a^** | **Adjusted^b^** |  |
|  | **n** | **%** |  | **n** | **%** |  |  |  |  |  |
| **Death during hospitalizations** | 19 | 0.01 |  | 1,468 | 0.01 |  | 1.82 (0.67, 4.92) | 1.31 (0.42, 4.14) | 1.31 (0.42, 4.14) |  |
| **Cesarean delivery** | 62,496 | 38.05 |  | 7,718,434 | 33.06 |  | **1.24 (1.21, 1.28)** | **1.31 (1.28, 1.35)** | **1.32 (1.28, 1.35)** |  |
| **Length of stay >6 day** |  |  |  |  |  |  |  |  |  |  |
| **Vaginal birth** | 243 | 0.15 |  | 9,526 | 0.04 |  | **4.12 (3.02, 5.63)** | **4.03 (2.94, 5.52)** | **4.26 (3.10, 5.84)** |  |
| **Cesarean delivery** | 4,800 | 2.92 |  | 245,416 | 1.05 |  | **2.53 (2.35, 2.74)** | **2.46 (2.27, 2.66)** | **2.52 (2.33, 2.74)** |  |
| **Induction of labor** | 32,876 | 20.01 |  | 4,247,666 | 18.20 |  | **1.13 (1.09, 1.17)** | **1.05 (1.02, 1.09)** | **1.05 (1.02, 1.09)** |  |
| **Antepartum hemorrhage** | 4,138 | 2.52 |  | 356,821 | 1.53 |  | **1.67 (1.55, 1.79)** | **1.67 (1.55, 1.79)** | **1.67 (1.55, 1.79)** |  |
| **Placental abruption** | 3,127 | 1.90 |  | 246,768 | 1.06 |  | **1.82 (1.68, 1.97)** | **1.75 (1.62, 1.90)** | **1.75 (1.62, 1.90** |  |
| **Postpartum hemorrhage** | 5,460 | 3.32 |  | 654,043 | 2.80 |  | **1.19 (1.11, 1.28)** | **1.22 (1.13, 1.31)** | **1.22 (1.13, 1.31)** |  |
| **Spontaneous delivery <37-week gestation** | 20,059 | 12.21 |  | 1,692,651 | 7.25 |  | **1.78 (1.71, 1.85)** | **1.75 (1.68, 1.82)** | **1.79 (1.72, 1.86)** |  |
| **Stillbirth** | 1,811 | 1.10 |  | 153,045 | 0.66 |  | **1.69 (1.52, 1.88)** | **1.64 (1.47, 1.82)** | **1.64 (1.47, 1.82)** |  |
| **Premature rupture of membranes** | 8,318 | 5.06 |  | 904,679 | 3.88 |  | **1.32 (1.25, 1.40)** | **1.31 (1.24, 1.39)** | **1.31 (1.24, 1.39)** |  |
| **Excessive fetal growth** | 3,807 | 2.32 |  | 609,142 | 2.61 |  | **0.89 (0.82, 0.96)** | **0.91 (0.85, 0.98)** | **0.91 (0.85, 0.98)** |  |
| **Poor Fetal growth** | 6,668 | 4.06 |  | 507,269 | 2.17 |  | **1.91 (1.79, 2.03)** | **1.75 (1.65, 1.86)** | **1.75 (1.65, 1.86)** |  |
| **Fetal distress** | 28,282 | 17.22 |  | 3,342,430 | 14.32 |  | **1.25 (1.20, 1.29)** | **1.21 (1.17, 1.26)** | **1.21 (1.17, 1.26)** |  |
| **Fetal abnormalities** | 4,380 | 2.67 |  | 337,978 | 1.45 |  | **1.87 (1.73, 2.01)** | **1.83 (1.70, 1.97)** | **1.83 (1.70, 1.97)** |  |

Abbreviations: SE, standard error; OR, odds ratio; CI, confidence interval

^a^ Adjusted for maternal age (continuous), race, median household income quartiles, hospital location, hospital region, and year

^b^ Further adjusted for multiple birth

**Supplement Table 4. Obstetric and neonatal outcomes among women with and without psychosis during singleton delivery hospitalizations (N = 23,076,251)**

| **Obstetric and neonatal outcomes** | **Women** | | | | |  | **OR (95% CI)** | |
| --- | --- | --- | --- | --- | --- | --- | --- | --- |
|  | **With psychosis**  **(N =** 161186**)** | |  | **Without psychosis**  **(N =** 22915065**)** | |  | **Unadjusted** | **Adjusted^a^** |
|  | **n** | **%** |  | **n** | **%** |  |  |  |
| **Death during hospitalizations** | 19 |  |  | 1,437 |  |  | 1.37 (0.44, 4.32) | 1.34 (0.43, 4.24) |
| **Cesarean delivery** | 60057 |  |  | 7394537 |  |  | **1.25 (1.21, 1.28)** | **1.32 (1.28, 1.35)** |
| **Length of stay >6 day** |  |  |  |  |  |  |  |  |
| **Vaginal birth** | 234 |  |  | 8566 |  |  | **4.42 (3.22, 6.06)** | **4.27 (3.10, 5.88)** |
| **Cesarean delivery** | 4321 |  |  | 203256 |  |  | **2.73 (2.52, 2.96)** | **2.65 (2.44, 2.87)** |
| **Induction of labor** | 32876 |  |  | 4247666 |  |  | **1.13 (1.09, 1.17)** | **1.05 (1.02, 1.09)** |
| **Antepartum hemorrhage** | 3988 |  |  | 343163 |  |  | **1.67 (1.55, 1.79)** | **1.67 (1.55, 1.79)** |
| **Placental abruption** | 3007 |  |  | 237372 |  |  | **1.81 (1.67, 1.96)** | **1.75 (1.61, 1.90)** |
| **Postpartum hemorrhage** | 5306 |  |  | 628125 |  |  | **1.22 (1.14, 1.31)** | **1.23 (1.14, 1.32)** |
| **Spontaneous delivery <37-week gestation** | 18342 |  |  | 1496179 |  |  | **1.84 (1.77, 1.92)** | **1.81 (1.74, 1.88)** |
| **Stillbirth** | 1655 |  |  | 137489 |  |  | **1.71 (1.54, 1.90)** | **1.66 (1.49, 1.85)** |
| **Premature rupture of membranes** | 7982 |  |  | 864586 |  |  | **1.33 (1.26, 1.41)** | **1.32 (1.24, 1.40)** |
| **Excessive fetal growth** | 3802 |  |  | 608295 |  |  | **0.89 (0.82, 0.96)** | **0.91 (0.85, 0.98)** |
| **Poor Fetal growth** | 6318 |  |  | 470897 |  |  | **1.95 (1.83, 2.08)** | **1.78 (1.67, 1.89)** |
| **Fetal distress** | 27933 |  |  | 3299666 |  |  | **1.25 (1.20, 1.30)** | **1.21 (1.17, 1.26)** |
| **Fetal abnormalities** | 4256 |  |  | 325751 |  |  | **1.88 (1.74, 2.03)** | **1.84 (1.71, 1.99)** |

Abbreviations: SE, standard error; OR, odds ratio; CI, confidence interval

^a^ Adjusted for maternal age (continuous), race, median household income quartiles, hospital location, hospital region, and year
